# Incidence and management of CAR-T neurotoxicity in patients with multiple myeloma treated with ciltacabtagene autoleucel in CARTITUDE studies

**DOI:** 10.1038/s41408-022-00629-1

**Published:** 2022-02-24

**Authors:** Adam D. Cohen, Samir Parekh, Bianca D. Santomasso, Jaime Gállego Pérez-Larraya, Niels W. C. J. van de Donk, Bertrand Arnulf, Maria-Victoria Mateos, Nikoletta Lendvai, Carolyn C. Jackson, Kevin C. De Braganca, Jordan M. Schecter, Loreta Marquez, Erin Lee, Ingrid Cornax, Enrique Zudaire, Claire Li, Yunsi Olyslager, Deepu Madduri, Helen Varsos, Lida Pacaud, Muhammad Akram, Dong Geng, Andrzej Jakubowiak, Hermann Einsele, Sundar Jagannath

**Affiliations:** 1 Abramson Cancer Center, University of Pennsylvania, Philadelphia, PA USA; 2 Mount Sinai Medical Center, New York, NY USA; 3 Department of Neurology, Memorial Sloan Kettering Cancer Center, New York, NY USA; 4 Department of Neurology, Clínica Universidad de Navarra, Pamplona, Spain; 5 Department of Hematology, Amsterdam University Medical Center, Vrije Universiteit Amsterdam, Cancer Center Amsterdam, Amsterdam, The Netherlands; 6 Hôpital Saint Louis, Paris, France; 7 University Hospital of Salamanca/Instituto de Investigación Biomédica de Salamanca, Salamanca, Spain; 8 Janssen R&D, Raritan, NJ USA; 9 Janssen R&D, Spring House, PA USA; 10 Janssen R&D, Beerse, Belgium; 11 Legend Biotech USA, Inc, Piscataway, NJ USA; 12 University of Chicago, Chicago, IL USA; 13 Universitätsklinikum Würzburg, Medizinische Klinik und Poliklinik II, Würzburg, Germany

**Keywords:** Targeted therapies, Myeloma

## Abstract

Chimeric antigen receptor (CAR) T-cell therapies are highly effective for multiple myeloma (MM) but their impressive efficacy is associated with treatment-related neurotoxicities in some patients. In CARTITUDE-1, 5% of patients with MM reported movement and neurocognitive treatment-emergent adverse events (MNTs) with ciltacabtagene autoleucel (cilta-cel), a B-cell maturation antigen-targeted CAR T-cell therapy. We assessed the associated factors for MNTs in CARTITUDE-1. Based on common features, patients who experienced MNTs were characterized by the presence of a combination of at least two variables: high tumor burden, grade ≥2 cytokine release syndrome (CRS) or any grade immune effector cell-associated neurotoxicity syndrome (ICANS) after cilta-cel infusion, and high CAR T-cell expansion/persistence. Strategies were implemented across the cilta-cel development program to monitor and manage patients with MNTs, including enhanced bridging therapy to reduce baseline tumor burden, early aggressive treatment of CRS and ICANS, handwriting assessments for early symptom detection, and extended monitoring/reporting time for neurotoxicity beyond 100 days post-infusion. After successful implementation of these strategies, the incidence of MNTs was reduced from 5% to <1% across the cilta-cel program, supporting its favorable benefit–risk profile for treatment of MM.

## Introduction

Chimeric antigen receptor (CAR) T-cell therapies are novel and highly effective treatment approaches for various hematologic malignancies, including multiple myeloma (MM). The impressive remission rates with these therapies are sometimes accompanied by treatment-related neurotoxicities, which can be mild to life threatening [1–5]. Neurotoxicity events, including immune effector cell-associated neurotoxicity syndrome (ICANS), are heterogenous in nature with highly variable clinical presentation. Symptoms of ICANS may include aphasia, altered consciousness, cognitive skills impairment, motor weakness, seizures, and cerebral edema [6, 7]. Other neurotoxicity events, usually with symptoms that do not fit the current definition for ICANS, have also been described with CAR T-cell therapies [8, 9].

The pathophysiology of neurotoxicities remains to be fully elucidated. ICANS can occur concurrently with cytokine release syndrome (CRS), a common side effect of CAR T-cell therapies, or days after resolution of CRS. The time to neurotoxicity onset after CAR T-cell infusion also varies; some studies have reported an onset within 3–4 weeks of treatment [7, 10], whereas others have shown delayed events arising >4 weeks post treatment [2, 11]. Thus, CAR T-cell–related neurotoxicities can have a highly variable course that requires careful monitoring and timely management to avoid potentially life-threatening or permanent neurologic sequelae.

Ciltacabtagene autoleucel (cilta-cel), a CAR T-cell therapy expressing two B-cell maturation antigen (BCMA)-targeting single binding domains, demonstrated deep and durable responses in heavily pretreated patients with relapsed/refractory MM in the CARTITUDE-1 phase 1b/2 study [12, 13]. Overall response rate was 98%, with 80% of patients achieving stringent complete response [13]. After 18 months of follow-up, the progression-free survival and overall survival rates were 66% and 81%, respectively. Similar to other CAR T-cell therapies [1–4, 9, 14, 15], cilta-cel–treated patients experienced ICANS. Here, we report the incidence of other CAR T-cell neurotoxicities characterized by movement and neurocognitive treatment-emergent adverse events (MNTs) in CARTITUDE-1, which comprise a cluster of movement (e.g., micrographia, tremors), cognitive (e.g., memory loss, disturbance in attention), and personality changes (e.g. reduced facial expression, flat affect). Although some symptoms overlap with ICANS symptomatology (i.e., altered mental status, somnolence), MNT symptoms occur after a period of recovery from CRS and/or ICANS and may present in a unique pattern, including insidious onset; these symptoms are also generally non-responsive to steroids, often progressive and have longer duration than ICANS. Of note, patients also had normal to near normal ICE scores at the time of MNT presentation, which is inconsistent with the current literature definition of ICANS [6]. Possible associated factors, underlying pathology, patient management strategies, and implications for clinical practice are presented.

## Methods

### Study design and participants

Patients were enrolled in the open-label phase 1b/2 CARTITUDE-1 study (NCT03548207) in the USA (16 centers) and Japan (four centers). Full study design details and eligibility criteria have been previously described [12]. Briefly, eligible patients had a diagnosis of MM per International Myeloma Working Group criteria [16], received ≥3 prior lines of therapy or were double refractory to a proteasome inhibitor and an immunomodulatory drug, and had received a proteasome inhibitor, an immunomodulatory drug, and an anti-CD38 antibody. Patients had to have documented disease progression ≤12 months after the last line of therapy and were excluded if they had previously been treated with a CAR T-cell or BCMA-targeted therapy. Patients with known active or prior history of central nervous system involvement and those who exhibited clinical signs of meningeal involvement of MM were excluded from the study. If pre-existing disease was suspected at screening, neurology consultation and dedicated baseline neuroimaging were considered.

### Procedures

Apheresis was performed according to institutional standards, with a collection target of 6 × 10^9^ peripheral blood mononuclear cells (range, 2–20 × 10^9^). Bridging therapy was permitted when clinically indicated during the manufacturing process. Per protocol, patients could only receive short-term bridging therapy with agents they had previously been exposed to and that had generated at least a response or stable disease. Cilta-cel was given as a single infusion on Day 1 (target dose 0.75 × 10^6^ [range, 0.5–1.0 × 10^6^] CAR+ viable T cells/kg) 5–7 days after the start of lymphodepletion (cyclophosphamide 300 mg/m^2^ and fludarabine 30 mg/m^2^ daily for 3 days).

This study was conducted in accordance with the Declaration of Helsinki and International Conference on Harmonization Good Clinical Practice guidelines. All patients provided informed consent. The study protocol, amendments, and relevant documents were approved by an independent ethics committee/institutional review board at each study center.

### Assessments

Patients were monitored for safety and disease assessments from Day 1 to Day 100 post cilta-cel infusion and then every 28 days from Day 101 to study completion (2 years after the last patient was dosed). Survival status and subsequent anti-cancer therapy information were collected every 16 weeks following disease progression.

Adverse events were graded using National Cancer Institute Common Terminology Criteria for Adverse Events (NCI-CTCAE) version 5.0. In the combined analysis of CARTITUDE-1 phase 1b and phase 2, ICANS and CRS were graded by American Society for Transplantation and Cellular Therapy criteria [6], and other CAR T-cell neurotoxicities (onset after a period of recovery from CRS and ICANS) by NCI-CTCAE version 5.0.

In CARTITUDE-1, the cluster of symptoms that occurred with MNTs (a subset of other CAR T-cell neurotoxicities) were grouped into the following categories—movement disorder, cognitive impairment, and personality changes (Table 1). Patients were considered to have MNTs if they met all three of the following criteria: (i) must have reported at least one or more of the preferred terms in at least two of the above categories; (ii) these reported preferred terms must have occurred following the recovery of CRS and/or ICANS; and (iii) symptoms must have been assessed by the investigator as CAR T-cell–related neurotoxicity (but not recognized as ICANS).

**Table 1:** Movement and neurocognitive treatment-emergent adverse events in CARTITUDE-1.

Category	Preferred term
Movement disorder	Ataxia, Balance disorder, Bradykinesia, Cogwheel rigidity, Dysgraphia, Dyskinesia, Dysmetria, Essential tremor, Gait disturbance, Hand-eye coordination impaired, Micrographia, Motor dysfunction, Myoclonus, Parkinsonism, Posture abnormal, Resting tremor, Stereotypy, Tremor
Cognitive impairment	Amnesia, Apraxia, Bradyphrenia, Cognitive disorder, Confusional state, Depressed level of consciousness, Disturbance in attention, Encephalopathy, Incoherent, Leukoencephalopathy, Loss of consciousness, Memory impairment, Mental impairment, Mental status changes, Non-infective encephalitis, Psychomotor retardation
Personality changes	Flat affect, Personality change, Reduced facial expression

### Potential associated factors for MNTs

To determine the potential associated factors for MNTs, various parameters were evaluated including baseline demographics and disease characteristics; tumor burden; use of bridging therapies; viral infection prior to apheresis; prior radiotherapy of the brain; and selected baseline laboratory values of fibrinogen, C-reactive protein, ferritin, platelet counts, β-2 microglobulin, serum cytokines interleukin (IL)-6, IL-10, interferon (IFN)-γ, and serum soluble IL-2 receptor α, and estimated glomerular filtration rate.

Tumor burden is typically defined based on the degree of plasmacytosis; however, there is a lack of consensus on the definition. In this analysis, we defined tumor burden based not only on the degree of plasmacytosis but also the presence of paraprotein since bone marrow aspirates are subject to sampling error depending on the distribution of malignant plasma cells within the bone marrow space. Thus, patients were categorized as having high tumor burden at baseline (prior to lymphodepletion) if they had *any* of the following: plasma cell infiltrate in the bone marrow ≥80%, serum M-spike ≥5 g/dL, or involved serum free light chain ≥5000 mg/L. Those with low tumor burden had *all* of the following: plasma cell infiltrate in the bone marrow <50%, serum M-spike <3 g/dL, and serum free light chain <3000 mg/L. Patients who did not fit either criterion were categorized as having intermediate tumor burden.

Parameters evaluated post cilta-cel infusion were: CRS and ICANS (by grade), supportive therapies for CRS and ICANS, CAR T-cell expansion and persistence (defined as peripheral blood CAR-T cells *C*_max_ of >1000 cells/μL and CAR-T cells >300 cells/μL at Day 56), hematology values (absolute lymphocyte counts [ALC], absolute neutrophil counts, and platelet counts in the first 30 days), absolute CD4+ T-cell counts, absolute CAR+ T cells, frequency of CAR+ T cells, and cytokine levels (IL-6 and IFN-γ). Cerebrospinal fluid (CSF) samples of patients with MNTs were assessed for the presence of CAR+ T cells.

Chemistry, manufacturing, and controls assessment reviewed batches of the cilta-cel drug product used in patients with MNTs for any deviations that may have occurred during manufacturing.

### Monitoring and patient management strategies

A safety management team evaluated the cases of MNTs in CARTITUDE-1 to identify and implement various changes in the conduct of ongoing studies across the CARTITUDE program. Amendments were made to all the global protocols and patient informed consent forms with the aim to provide guidance to investigators at study sites.

### Statistical analysis

This analysis included the safety population from CARTITUDE-1 (all cilta-cel–treated patients). Descriptive statistics and frequency distribution with the numbers and percentages of patients in each category of the MNTs observed in CARTITUDE-1 were included as appropriate. Differences between patients with and without MNTs in the clinical variables were assessed using Wilcoxon rank sum test for continuous variables and Fisher’s exact test for categorical variables. Odds ratios and confidence intervals for logistic regression, with MNTs as a dependent variable, were used to evaluate each of the clinical variables as an independent variable.

## Results

Neurotoxicity data from a total of 97 patients in CARTITUDE-1 with a February 2021 cutoff date were included in this analysis, representing a median follow-up of 18 months. Detailed baseline characteristics of patients in CARTITUDE-1 have been previously published [12]. Patients’ median age was 61 years, 59% were male, and 24% had high-risk cytogenetic profile—defined as the presence of del17p, t(14:16), and t(4:14). Patients had received a median of six prior lines of therapy; 88% were triple-class refractory, 42% were penta-drug refractory, and 99% were refractory to their last line of therapy.

**Fig. 1:**
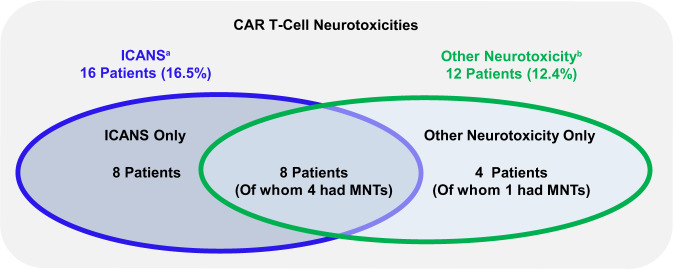
Overview of CAR T-cell neurotoxicities in CARTITUDE-1. ^a^ICANS per ASTCT consensus criteria. ^b^Neurotoxicity as assessed by the investigator to be related to cilta-cel occurring after a period of recovery from CRS and/or ICANS. ICANS and other neurotoxicity events are not mutually exclusive; eight (8.2%) patients experienced both ICANS and other neurotoxicity events of any grade. Patients in the other-neurotoxicity group (*n* = 12) include the subset with MNTs (*n* = 5). *ASTCT* American Society for Transplantation and Cellular Therapy, *CAR* chimeric antigen receptor, *CRS* cytokine release syndrome, *ICANS* immune effector cell-associated neurotoxicity syndrome, *MNTs* movement and neurocognitive treatment-emergent adverse events.

Total CAR T-cell neurotoxicities, including ICANS and other CAR T-cell neurotoxicities, were observed in 20.6% of patients; nine (9.3%) had grade 3/4 events and one (1.0%) had a grade 5 event. ICANS and other CAR T-cell neurotoxicities were not mutually exclusive as eight patients (8.2%) experienced both (Fig. 1). ICANS occurred in 16 (16%) patients, mostly grade 1/2 (14%) with one patient each having a grade 3 and 4 event; 15 of the 16 patients had concurrent CRS. Median time to onset of ICANS was 8 days (range, 3–12 days) and the median duration was 4 days (range, 1–12). All patients had recovered from ICANS at data cutoff. Other CAR T-cell neurotoxicities occurred in 12 (12.4%) patients; median onset was 26.5 days (range, 11–108) after cilta-cel infusion, and median time to resolution was 70 days (range, 2–159). Symptoms associated with other neurotoxicities were variable and wide ranging and have been previously described [12]. Of these 12 patients, five experienced MNTs as described below. Neurotoxicity in the remaining seven patients manifested as facial paralysis (*n* = 1), neurotoxicity (*n* = 1), concentration impairment (*n* = 1), diplopia (*n* = 1), cranial nerve palsy (*n* = 1), sensory loss, ataxia, peripheral motor neuropathy, and peripheral sensory neuropathy (all in one patient), and altered mental status and nystagmus (both in one patient; Supplementary Table 1). Five of these 7 patients recovered from neurotoxicity, and 2 died of other causes.

### MNTs in CARTITUDE-1 and potential associated factors

Five patients experienced MNTs, which included a cluster of movement, cognitive, and personality changes (Table 1). Details of these patients are described in Table 2. One patient experienced a grade 2 event, three patients experienced grade 3 events, and one patient experienced a grade 5 event; median time to onset was 27.0 days. The median onset of MNTs was 17 days (range, 3−94) after the recovery of CRS and ICANS. Supportive measures for MNTs included steroids, systemic chemotherapy (cyclophosphamide), intrathecal chemotherapy (methotrexate, cytarabine), anakinra, dasatinib, siltuximab, and other agents (e.g., carbidopa/levodopa, levetiracetam) with limited or no observed improvement in symptoms. Three of the five patients with MNTs achieved stringent complete response and two achieved very good partial response, as assessed by an independent review committee, following treatment with cilta-cel.

**Table 2:** Characteristics of MNTs in CARTITUDE-1.

Characteristic	*N* = 97
Patients with MNT^a^, *n* (%)	5 (5.2)
Maximum toxicity grade, *n* *(%)*	
Grade 1	0
Grade 2	1 (1.0)
Grade 3	3 (3.1)
Grade 4	0
Grade 5	1 (1.0)
Median time to onset, days (range)	27.0 (14–108)
Outcome of neurotoxic event, n (%)	
Recovered or resolved	0
Not recovered or not resolved	3 (3.1)^b^
Recovering or resolving	1 (1.0)
Fatal	1 (1.0)

*ICANS* immune effector cell-associated neurotoxicity syndrome, *MNTs* movement and neurocognitive treatment-emergent adverse events.

^a^Events not reported as ICANS (i.e., onset after a period of recovery from cytokine release syndrome and ICANS).

^b^Not recovered or not resolved at the time of data cutoff; two of these patients died due to other causes (one due to septic shock and one due to lung abscess).

Baseline and post-infusion characteristics in patients with and without MNTs are shown in Table 3. All five patients with MNTs were male and had received prior bridging therapy; three (60%) had high tumor burden at baseline. Post cilta-cel infusion, all five patients developed grade ≥2 CRS; four (80%) had ICANS, and four (80%) had high CAR T-cell expansion and persistence. Of the 92 patients without MNTs, 13 (14.1%) had high tumor burden, 38 (41.3%) had grade ≥2 CRS, 12 (13.0%) had ICANS, and eight (8.7%) had high CAR T-cell expansion and persistence.

**Table 3:** Baseline and post-infusion characteristics in patients with and without MNTs in CARTITUDE-1.

Baseline characteristic	MNTs	
	No (*n* = 92)	Yes (*n* = 5)
Age, years; *n* (%)		
<65	59 (64.1)	3 (60.0)
65–75	26 (28.3)	1 (20.0)
>75	7 (7.6)	1 (20.0)
Sex, *n* (%)		
Female	40 (43.5)	0
Male	52 (56.5)	5 (100.0)
Race, *n* (%)		
White	64 (69.6)	5 (100.0)
African American	17 (18.5)	0
Other	11 (12.0)	0
Ethnicity, *n* (%)		
Hispanic or Latino	6 (6.5)	0
Non-Hispanic or Latino	80 (87.0)	5 (100.0)
Not reported	6 (6.5)	0
ECOG PS score^a^, *n* (%)		
0	36 (39.1)	3 (60.0)
1	52 (56.5)	2 (40.0)
2	4 (4.3)	0
Tumor burden category^b^, *n* (%)		
High	13 (14.1)	3 (60.0)
Intermediate	21 (22.8)	1 (20.0)
Low	58 (63.0)	1 (20.0)
Type of myeloma, *n* (%)		
IgG	55 (59.8)	2 (40.0)
Non-IgG	37 (40.2)	3 (60.0)
Measurable disease type, *n* (%)		
Serum only, serum and urine	51 (55.4)	4 (80.0)
Urine only, FLC, not evaluable	41 (44.6)	1 (20.0)^c^
Extramedullary plasmacytoma, *n* (%)		
No	80 (87.0)	4 (80.0)
Yes	12 (13.0)	1 (20.0)
Bone-based plasmacytoma, *n* (%)		
No	87 (94.6)	4 (80.0)
Yes	5 (5.4)	1 (20.0)
Number of prior lines of therapy, *n* (%)		
3–5	46 (50.0)	3 (60.0)
≥6	46 (50.0)	2 (40.0)
Prior radiotherapy including brain area, *n* (%)		
No	84 (91.3)	5 (100.0)
Yes	8 (8.7)	0
Prior bridging therapy, *n* (%)		
No	24 (26.1)	0
Yes	68 (73.9)	5 (100.0)
Type of prior bridging therapy, *n* (%)		
Daratumumab		
No	78 (84.8)	4 (80.0)
Yes	14 (15.2)	1 (20.0)
Lenalidomide		
No	86 (93.5)	5 (100.0)
Yes	6 (6.5)	0
**Post-infusion characteristic**		
Total CAR + viable T-cells infused (×10^6^), *n* (%)		
<median value	46 (50.0)	2 (40.0)
≥ median value	46 (50.0)	3 (60.0)
High cell expansion/persistence^d^, *n* (%)		
No	84 (91.3)	1 (20.0)
Yes	8 (8.7)	4 (80.0)
CRS, *n* (%)		
No	5 (5.4)	0
Yes	87 (94.6)	5 (100.0)
CRS maximum toxicity grade, *n* (%)		
Grade <2	54 (58.7)	0
Grade ≥2	38 (41.3)	5 (100.0)
ICANS, *n* (%)		
No	80 (87.0)	1 (20.0)
Yes	12 (13.0)	4 (80.0)

*CAR* chimeric antigen receptor, *C*_max_ maximum CAR transgene systemic level, *CRS* cytokine release syndrome, *ECOG PS* Eastern Cooperative Oncology Group performance status, *FLC* free light chain, *ICANS* immune effector cell-associated neurotoxicity syndrome, *IgG* immunoglobulin G, *MNTs* movement and neurocognitive treatment-emergent adverse events.

^a^The last non-missing ECOG PS score on or prior to date of cilta-cel infusion was used; all patients met the inclusion criteria of ECOG PS score of 0 or 1 during screening.

^b^Patients were categorized as having high tumor burden at baseline (prior to lymphodepletion) if they met any of the following: plasma cell infiltrate in the bone marrow ≥80%, serum M-spike ≥5 g/dL, serum free light chain ≥5000 mg/L. Those with low tumor burden had all of the following: plasma cell infiltrate in the bone marrow <50%, serum M-spike <3 g/dL, and serum free light chain <3000 mg/L. Patients who did not fit either criterion were categorized as having intermediate tumor burden.

^c^Patient was FLC-evaluable.

^d^Patients with peripheral blood CAR T cells *C*_max_ of >1000 cells/μL and CAR T cells >300 cells/μL at Day 56.

**Fig. 2:**
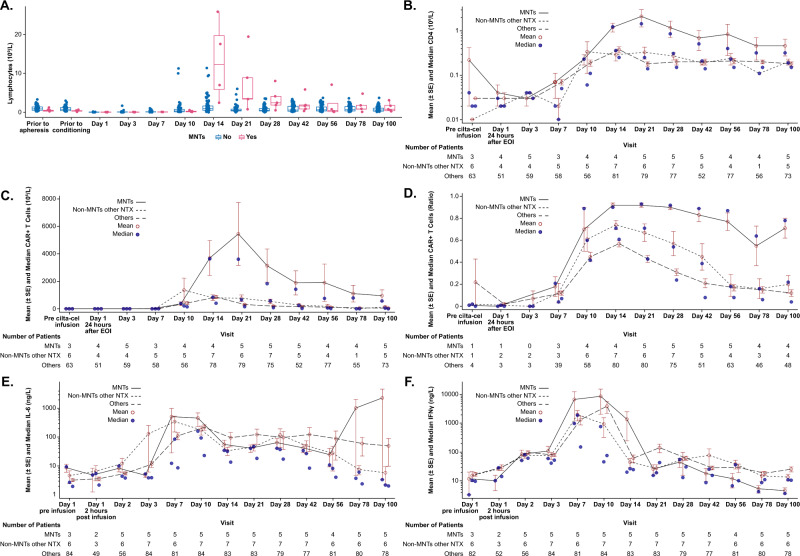
Analysis of lymphocytes, T cells, and cytokines over time following cilta-cel infusion in patients with and without MNTs in CARTITUDE-1. Values are shown for **A** lymphocyte counts; **B** absolute CD4+ T-cell counts; **C** absolute CAR+ T cells; **D** frequency of CAR+ T cells; **E** IL-6; and **F** IFN-γ. *CAR* chimeric antigen receptor, *EOI* end of infusion, *IFN* interferon*,* *IL* interleukin, *MNT(s)* movement and neurocognitive treatment-emergent adverse event(s), *NTX* neurotoxicity, *SE* standard error.

**Fig. 3:**
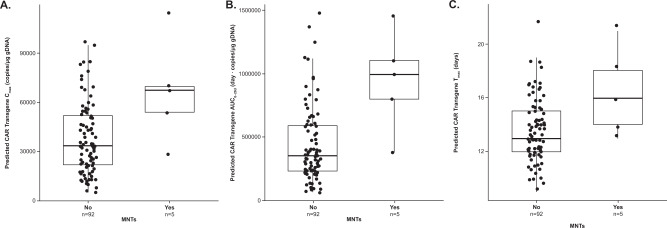
Analysis of CAR transgene levels in patients with and without MNTs in CARTITUDE-1. Predicted values are shown for **A** *C*_max_, **B** *AUC*_0–28d_, and **C** *T*_max_. *AUC*_0–28d_ area under the CAR transgene systemic level-time curve from the first dose to Day 28, *CAR* chimeric antigen receptor, *C*_max_ maximum CAR transgene systemic level, *gDNA* genomic DNA, *MNTs* movement and neurocognitive treatment-emergent adverse events, *T*_max_ time of maximum cilta-cel transgene expansion.

Analysis of clinical laboratory values showed higher ALC, absolute CD4+ T cells, and absolute CAR+ T cells on Days 14, 21, and 28 post cilta-cel infusion in patients with MNTs than in those without MNTs (Fig. 2A–C). From Day 14 to Day 100 post cilta-cel infusion, the frequency of CAR+ T cells was higher in patients with MNTs compared with those who had other neurotoxicities without MNTs and those who had neither (Fig. 2D). Similarly, peak levels of IL-6 and IFN-γ were higher in patients with MNTs than in the other groups following treatment with cilta-cel (Fig. 2E and F). Pharmacokinetic analysis of CAR transgene levels in peripheral blood of patients with MNTs showed high CAR T-cell peak expansion and a trend of delayed *T*_max_ and high overall exposure (expansion and persistence) (Fig. 3). CSF examinations in two patients with MNTs for whom CSF samples were available showed CAR+ T cells.

**Fig. 4:**
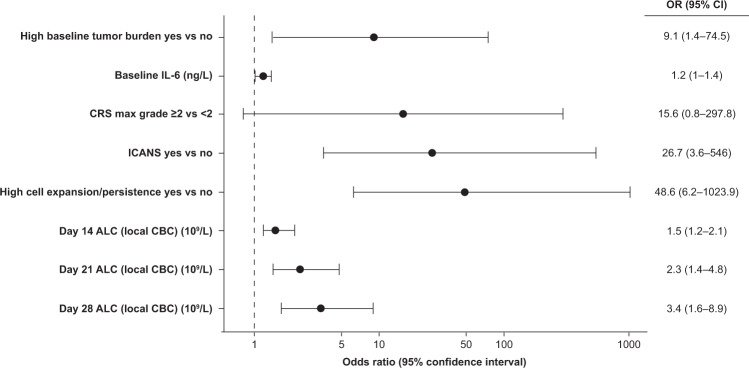
Forest plot of potential factors associated with MNTs in CARTITUDE-1. *ALC* absolute lymphocyte count, *CBC* complete blood count, *CRS* cytokine release syndrome, *ICANS* immune effector cell-associated neurotoxicity syndrome, *IL* interleukin, *MNTs* movement and neurocognitive treatment-emergent adverse events.

Logistic regression analysis of odds ratios for potential associated factors showed an association between the occurrence of MNTs and high tumor burden at baseline before the start of lymphodepletion, high levels of IL-6 at baseline, grade ≥2 CRS, incidence of ICANS, high CAR T-cell expansion and persistence, and high ALC (including absolute CD4+ T cells) on Days 14, 21, and 28 post cilta-cel infusion (Fig. 4).

As noted above, a trend toward higher IL-6 levels at baseline was seen in patients with MNTs versus those without, but there were no relevant differences in baseline levels of IL-10, IFN-γ, or IL-2 receptor α between patients with and without MNTs (Supplementary Fig. 1). The CAR+ T-cell CD4/CD8 ratio was similar in patients with or without MNTs (Supplementary Fig. 2). Memory T-cell subsets at apheresis showed no clear differences among patients with ICANS, other CAR T-cell neurotoxicities, or MNTs (Supplementary Fig. 3). Similarly, treatment dose–response relationship showed no apparent trend between the infused cilta-cel dose (0.75 × 10^6^ [range, 0.5–1.0 × 10^6^] CAR+ viable T cells/kg) and other CAR T-cell neurotoxicities (Supplementary Fig. 4).

Chemistry, manufacturing, and controls assessment of the manufacturing process of CAR-T vector batches for the five patients with MNTs showed no relevant deviations in the manufacturing process. Furthermore, there were no notable product characteristics that would have distinguished patients who had MNTs from those who did not experience these events (data not shown).

Of the five patients with MNTs in CARTITUDE-1, one had ongoing symptoms at the time of data cutoff, one was recovering/resolving, and three died. The causes of death were lung abscess, septic shock, and neurotoxicity on Days 119, 162, and 247 post cilta-cel infusion, respectively. All three fatal events were considered to be treatment-related by the investigator. Neuropathology findings from the two patients who had autopsy showed focal gliosis and T-cell infiltrate (CD8+>CD4+) in the basal ganglia; it is unknown if these were CAR+ T cells. No abnormalities were reported in other brain regions (i.e., cerebellum, substantia nigra) potentially associated with MNTs, and there was preservation of pigmentation in the substantia nigra. In one of the two patients, while brain magnetic resonance imaging and head computed tomography scans did not demonstrate any significant abnormalities, FDG-PET scan showed hypometabolism in the basal ganglia. A dopamine uptake scan was negative, indicating normal dopamine signaling.

### Monitoring and patient-management strategies

**Fig. 5:**
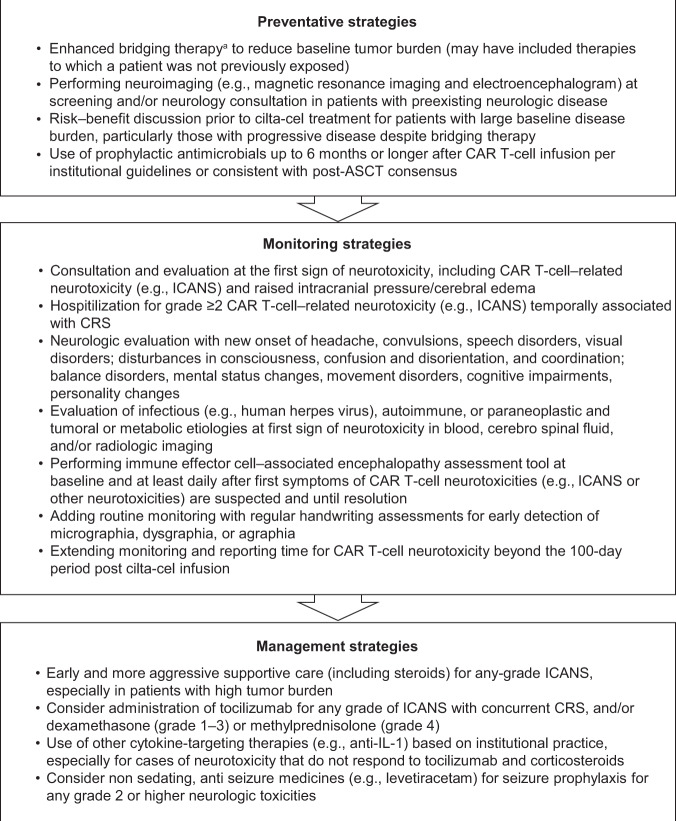
Patient-management strategies for neurologic adverse events following treatment with cilta-cel. ^a^Fewer restrictions (per protocol) on the investigator’s choice of bridging therapy, including its duration, to reduce baseline tumor burden before cilta-cel infusion. *ASTCT* American Society for Transplantation and Cellular Therapy, *CAR* chimeric antigen receptor, *CRS* cytokine release syndrome, *ICANS* immune effector cell-associated neurotoxicity syndrome, *IL* interleukin.

To minimize risk for MNTs, several preventive, monitoring, and patient management strategies were implemented across all ongoing studies in the cilta-cel clinical development program based on the results described above (Fig. 5). Preventive measures included fewer restrictions per protocol on the investigator’s choice of bridging therapy used, including its duration, to reduce baseline tumor burden before cilta-cel infusion (i.e., enhanced bridging therapy based on the investigator’s choice), and a risk–benefit discussion for patients with high baseline disease burden, particularly in those with progressive disease despite bridging therapy. Monitoring strategies included handwriting assessments using a novel handwriting tool for early detection of neurotoxicity symptoms (Supplementary Table 2), and extended monitoring and reporting time for CAR T-cell neurotoxicities up to 1 year after cilta-cel infusion. Management strategies included early and aggressive supportive care including steroids for any-grade ICANS, especially in patients with high tumor burden, and tocilizumab for any-grade ICANS with concurrent CRS. Guidance for physicians on management included potential diagnostics for new neurologic or psychiatric symptoms (see Supplementary Table 3).

### MNTs in ongoing trials with cilta-cel

More than 150 patients were subsequently dosed across the cilta-cel clinical development program since the implementation of monitoring and patient management strategies as described above.

As of the April 15, 2021 data cutoff, one patient in the multicohort phase 2 CARTITUDE-2 study (NCT04133636) developed other neurotoxicity, presenting with a cluster of symptoms characteristic of MNTs. As previously described, CARTITUDE-2 is evaluating cilta-cel safety and efficacy in various clinical settings for patients with MM [17]. This patient (male; 44 years of age), who was enrolled in cohort B (one prior line of therapy, early relapse), had characteristics that were identified among patients in CARTITUDE-1 as associated factors for MNTs such as high tumor burden at baseline, worsening disease burden despite bridging therapy, development of grade 4 CRS, and high CAR T-cell expansion and persistence. ALC on Day 21 post cilta-cel infusion was 8.4 × 10^9^/L; CD4+ T-cell count on Day 14 was not available for this patient. Micrographia was one of the first neurologic manifestations of MNTs in this patient. The patient also developed variable and wide-ranging symptoms associated with these neurotoxicity events and had a cluster of MNTs including bradykinesia, rigidity, bradyphrenia, flat effect, apathy, gait disorder, and cognitive impairment which began on Day 38 after cilta-cel infusion. After treatment with high-dose methylprednisolone, plasmapheresis, and intravenous immunoglobulin, the patient was reported to be stable with mild improvement at data cutoff, and had achieved very good partial response as best response to treatment with cilta-cel.

## Discussion

A single infusion of cilta-cel resulted in unprecedented efficacy in heavily pretreated patients with MM in the CARTITUDE-1 study [12]. Responses were early, durable, and deepened over time, and the highest (overall response rate, 98%; stringent complete response, 80%) achieved to date in this patient population [12, 13]. Responses were maintained in an estimated 66% of patients with no disease progression or death for 18 months after cilta-cel administration. Considerations of the benefits of cilta-cel treatment must also be balanced by potential risks. CAR T-cell-related neurotoxicities, including ICANS and other CAR T-cell neurotoxicities, occurred in 20 (21%) cilta-cel-treated patients in CARTITUDE-1. Five (5%) of these patients presented with neurotoxicities beyond ICANS that were characterized by a cluster of MNTs. Symptoms were wide-ranging, included movement, cognitive, and personality changes, and occurred after a period of recovery from CRS and/or ICANS. All five patients had grade ≥2 CRS and most had ICANS post cilta-cel infusion. Based on common features among these patients, potential associated factors were identified: a combination of at least two variables such as high tumor burden, grade ≥2 CRS post-infusion, ICANS post-infusion, and high CAR T-cell expansion and persistence. Other associated factors included high baseline IL-6, high lymphocyte counts on Days 14, 21, and 28 post-infusion, and high peak levels of IL-6 and IFN-γ in peripheral blood. There were no relevant deviations in the manufacturing process of the CAR-T vector batches for the five patients with MNTs.

Similar to other CAR T-cell therapies [1–4, 9, 14, 15], cilta-cel–treated patients experienced ICANS. As of June 30, 2021, a total of 1374 cases of neurotoxicity, including ICANS, were recorded in the US Food and Drug Administration Adverse Event Reporting System for the three approved anti-CD19 CAR T-cell therapies for hematologic cancers [5]. These neurotoxicities had a wide range of symptoms, with varying time to onset and duration, including headache, encephalopathy, tremor, confusional state, delirium, agitation, somnolence, sleep disorders, peripheral neuropathy, parkinsonism, rigidity/cogwheeling, and ataxia [2–4, 7, 9, 14, 15, 18]. Neurotoxicities were observed with BCMA-targeting anti-myeloma CAR T-cell therapies at rates ranging from 13% to 44% [1, 8, 19–21]. Additionally, symptoms characteristic of MNTs (i.e., grade 3 parkinsonism) were reported with the approved anti-BCMA CAR-T therapy, idecabtagene vicleucel, in another study in MM [20]. Although MNTs are numerically higher in CARTITUDE-1, similarities in the type of neurologic events across various CAR T-cell therapies indicate this phenomenon may be attributable to a class effect. We investigated BCMA expression in normal brain as a potential explanation for this class effect but found no detectable immunoreactivity to BCMA protein in the brain samples of normal adults [22], which precluded any determination of a relationship between BCMA expression and incidence of MNTs but does not preclude aberrant expression in selected areas of the brain.

The underlying pathophysiology of neurotoxicities with CAR T-cell therapies is poorly understood. Reports in the literature suggest markers for neurotoxicity associated with BCMA-directed CAR T-cell therapies are similar to those with CD19-directed immunotherapies, and have demonstrated a correlation of ICANS with the presence and severity of CRS, and high tumor burden [7, 8, 10, 23, 24]. In one study of patients treated with CD19-directed CAR T-cell therapy, CSF levels of S100 calcium-binding protein B and glial fibrillary acidic protein increased during ICANS, indicating astrocyte injury [25]. Other studies have proposed that increases in the levels of IL-6, IFN-γ, and TNF-α from CAR T cells may induce endothelial activation, which may in turn disrupt the blood–brain barrier and lead to development of ICANS [7, 10]. Although the clinical presentation of MNTs overlaps with Parkinson’s disease, neuropathology findings in the two patients with MNTs in CARTITUDE-1 for whom autopsies were available showed intact substantia nigra, suggesting a distinct pathophysiology of these events. These autopsy findings were consistent with a negative dopamine uptake scan in one patient and lack of response to treatment with carbidopa/levodopa in both patients, providing further evidence of a pathophysiology that is distinct from Parkinson’s disease.

In the absence of well-documented predictors of neurotoxicities in the literature, vigilant monitoring is recommended for prospective patients treated with CAR T-cell therapies, and is critical for early detection, adequate management, and minimization of morbidity. This was the rationale for the development and implementation of patient management strategies to minimize the incidence of CAR T-cell neurotoxicities during the CARTITUDE clinical trials. Guidance for physicians includes recommendations for enhanced bridging therapy to reduce baseline tumor burden, early aggressive treatment of CRS and ICANS, handwriting assessments for early detection of neurotoxicity symptoms, and extended monitoring and reporting time for CAR T-cell neurotoxicity beyond 100 days post-cilta-cel infusion. The success of monitoring and patient management strategies was demonstrated by the reduced incidence of MNTs after their implementation in the cilta-cel program as of the cutoff date for this report. With over 150 patients subsequently dosed across the cilta-cel clinical development program, the overall incidence of MNTs decreased from 5% to <1%.

While high CAR T-cell expansion, persistence, or frequency have emerged as potential factors associated with MNTs, the utility of these parameters in real-world clinical settings is limited. CAR-T levels are often difficult to measure, and symptoms of MNTs may manifest in the patient before any evidence of prolonged CAR T-cell persistence. In our analysis, elevated levels of CD4+ T cells (>1000 × 10^6^/L) on Day 14 post-infusion, and higher ALC (>3 × 10^9^/L) on ~Day 21 post-infusion appear to correlate with a higher risk of developing MNTs, particularly in men. Hence, these biomarkers (independent of each other) may be useful in routine clinical practice as they can be assessed rapidly and before prolonged CAR T-cell persistence is detected. As expected, flow cytometry analysis of lymphocytes between Day 14 and Day 28 post-infusion showed a T-cell population primarily composed of CAR+ T cells. This suggests a potential for the utility of CD4+ T cells on Day 14 and ALC on Day 21 post-infusion as surrogate markers for CAR-T levels. However, our findings show not all patients with ALC > 3 × 10^9^/L on Day 21 post-infusion develop MNTs. Therefore, this hypothesis warrants further validation in a larger cohort given that in over 150 patients subsequently treated with cilta-cel, only one developed MNTs. Notably, this patient had an ALC of 8.4 × 10^9^/L on Day 21 post-infusion; CD4+ T-cell count on Day 14 post-infusion was not available.

Limitations of our study should be noted. In CARTITUDE-1, the number of patients with MNTs was small; hence further analysis of associated factors for these toxicities is needed. As of the cutoff date for this report, the CARTITUDE-2 study is enrolling, and patient follow-up is ongoing; subsequent analyses of patients in various cohorts of this multicohort study will inform the safety profile of cilta-cel. Cilta-cel is being evaluated in earlier line settings in the phase 3 studies of CARTITUDE-4 (NCT04181827) and CARTITUDE-5 (NCT04923893), which will further inform the risk factors for CAR T-cell neurotoxicities, including MNTs.

Patients completing CARTITUDE-1 and other cilta-cel clinical trials will be enrolled in a long-term follow-up study for continued monitoring for up to 15 years. A planned observational post-authorization safety study will focus on areas including characterization and evaluation of identified risks, and long-term safety. Thus, the knowledge base about cilta-cel treatment of MM will continue to grow as data emerge on longer term efficacy and safety, mechanism of action, and patient perspectives.

## Supplementary information

Supplementary Appendix

## Data Availability

The data sharing policy of Janssen Pharmaceutical Companies of Johnson & Johnson is available at https://www.janssen.com/clinical-trials/transparency. As noted on this site, requests for access to the study data can be submitted through Yale Open Data Access (YODA) Project site at http://yoda.yale.edu.
