# Supplementary material for: Incidence and management of CAR-T neurotoxicity in patients with multiple myeloma treated with ciltacabtagene autoleucel in CARTITUDE studies
Source: Blood Cancer J. 2022 Feb 24;12(2):32. doi: 10.1038/s41408-022-00629-1 (PMC8873238; doi:10.1038/s41408-022-00629-1)

**Supplementary Appendix**

Supplement to: Cohen AD, Parekh S, Santomasso BD, et al. Incidence and management of CAR-T neurotoxicity in patients with multiple myeloma treated with ciltacabtagene autoleucel in CARTITUDE studies

**Supplementary Table 1.** Non-ICANS, non-MNT neurotoxicities.

| **Patient** | **Symptoms (Maximum grade)** | **Day of onset** | **Treatment** | **Resolution (duration of AE)** |
| --- | --- | --- | --- | --- |
| 1 | Facial paralysis (Grade 2) | Day 26 | Corticosteroids | Recovered/resolved  (70 days) |
| 2 | Neurotoxicity (Grade 4) | Day 93 | Anakinra, corticosteroids; anti-thymocyte immuoglobulin | Not recovered/resolved; patient died from respiratory failure on Day 121 post-infusion |
| 3 | Concentration impairment (Grade 2) | Day 28 | None | Recovered/resolved  (28 days) |
| 4 | Diplopia (Grade 3) | Day 11 | None | Recovered/resolved  (2 days) |
| 5 | Sensory loss (Grade 2)  Ataxia (Grade 2)  Peripheral motor neuropathy (Grade 3)  Peripheral sensory neuropathy (Grade 3) | Day 53  Day 74  Days 74 and 82  Days 74 and 127 | None | All recovered/resolved  (up to 138 days) |
| 6 | Cranial nerve palsy (Grade 3) | Days 21, 26, 79 | Corticosteroids | Recovered/resolved  (up to 54 days) |
| 7 | Altered mental status (grade 3)  Nystagmus (grade 2) | Day 17  Day 38 | Corticosteroids | Both not recovered/resolved; patient died from sepsis on Day 45 post-infusion |

**Supplementary Table 2.** Handwriting adverse event toxicity grading criteria.

| **Adverse event term** | **Grade 1** | **Grade 2** |
| --- | --- | --- |
| Micrographia:  Abnormally small or  cramped handwriting | Mildly smaller letters or reduced spacing (e.g., <50% decrease from baseline) | Moderately to severely smaller letters or reduced spacing (e.g., ≥50% decrease from baseline) |
| Dysgraphia:  Illegible writing or writing that takes an unusually long time or great effort | Mildly slower writing, impaired straightness of line, difficulty in completing task from baseline; most words are legible | Moderately to severely slower writing, impaired straightness of line, difficulty in completing task from baseline; most words are illegible |
| Agraphia:  Pathologic loss of the ability to write | Able to write part of a sentence (≥3 words); noted change from baseline | Able to write just 1 to 2 words, or unable to write any words; noted change from baseline |

**Supplementary Table 3.** Guidance for physicians for management of patients with neurologic or psychiatric symptoms following treatment with cilta-cel.

| **Symptom** | **Monitoring/management strategy** |
| --- | --- |
| - Movement disorder (e.g., micrographia or changes in handwriting, tremors, bradykinesia, rigidity, shuffling gait, impaired balance and coordination, difficulty writing, difficulty performing activities of daily living such as dressing or feeding oneself) - Cognitive impairments (e.g., memory loss or forgetfulness, disturbances in attention, mental slowness or fogginess, difficulty speaking or slurred speech, difficulty reading or understanding words) - Personality change (e.g., reduced facial expression, flat affect, reduced ability to express emotions, less communicative, disinterest in activities) | - Contact medical monitor - Refer the patient immediately to a neurologist for a full evaluation - Neurotoxicities should be monitored beyond 100 days post cilta-cel infusion - Potential diagnostics for patients with new neurologic symptoms - PET/CT of the brain and/or brain MRI with perfusion and an EEG - Lumbar puncture to rule out infection (in particular John Cunningham virus, herpes zoster virus, herpes simplex virus-1/2, HHV-6, HHV-7, Epstein-Barr virus, cytomegalovirus) - Molecular testing for HHV-6 and HHV-7 by polymerase chain reaction for viremia - CSF flow cytometry and cytology should be considered to rule out leptomeningeal disease - CSF analysis should be considered to rule out paraneoplastic syndromes - Thiamine level (consider empiric thiamine replacement while awaiting results) |

*CSF* cerebrospinal fluid, *EEG* electroencephalogram, *HHV* human herpes virus, *MRI* magnetic resonance imaging, *PET/CT* positron emission tomography/computed tomography.

**Supplementary Fig. 1 Cytokine levels at baseline in patients with and without MNTs in CARTITUDE-1.** Shown are baseline levels of **A** IL-6, **B** IL-10, **C** IFN-γ, and **D** IL-2 receptor α. *IFN* interferon, *IL* interleukin, *MNTs* movement and neurocognitive treatment-emergent adverse events.


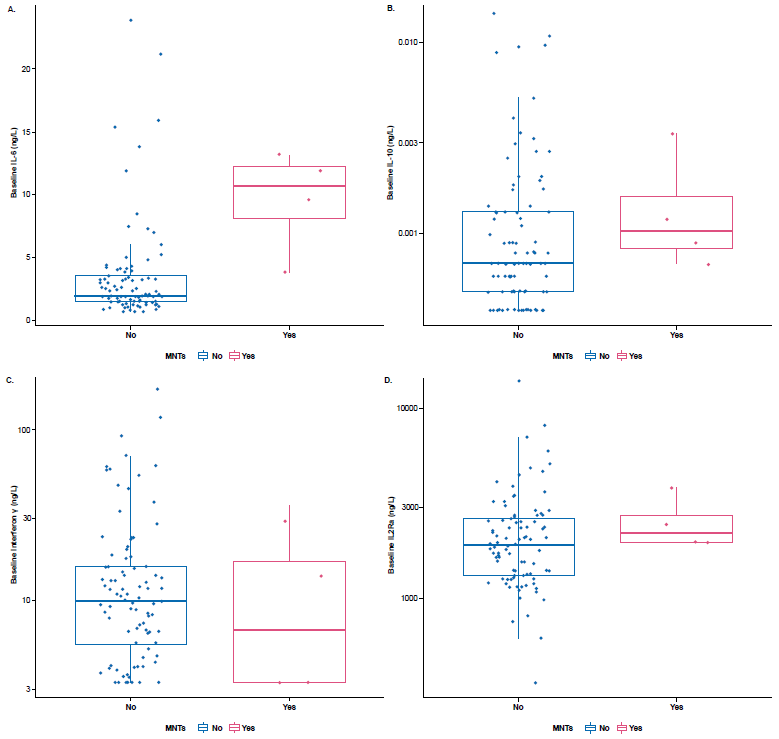


**Supplementary Fig. 2 CD4/CD8 CAR+ T-cell ratio^a^ in peripheral blood of patients with and without MNTs in CARTITUDE-1.** *CAR* chimeric antigen receptor, *MNTs* movement and neurocognitive treatment-emergent adverse events, *NTX* neurotoxicity, *T_max_* time of maximum concentration.

^a^At T_max_ of CD3+ CAR+ T cells in peripheral blood.


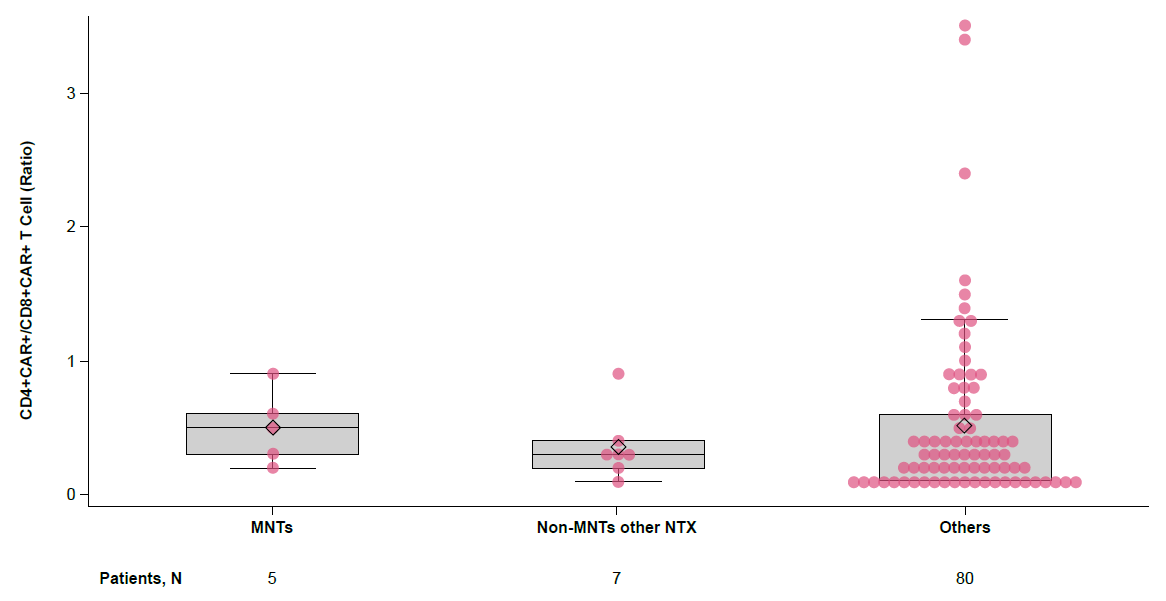


**Supplementary Fig. 3 T-cell memory phenotype at apheresis in patients with neurologic adverse events in CARTITUDE-1.** *Aph* apheresis, *CAR* chimeric antigen receptor, *ICANS* immune effector cell-associated neurotoxicity syndrome, *MNTs* movement and neurocognitive treatment-emergent adverse events, *NA* not applicable, *NTX* neurotoxicity, *Tcm* central memory, *Tem* effector memory, *Temra* effector memory re-expressing CD45RA, *Tn* naïve, *Tscm* stem cell memory.


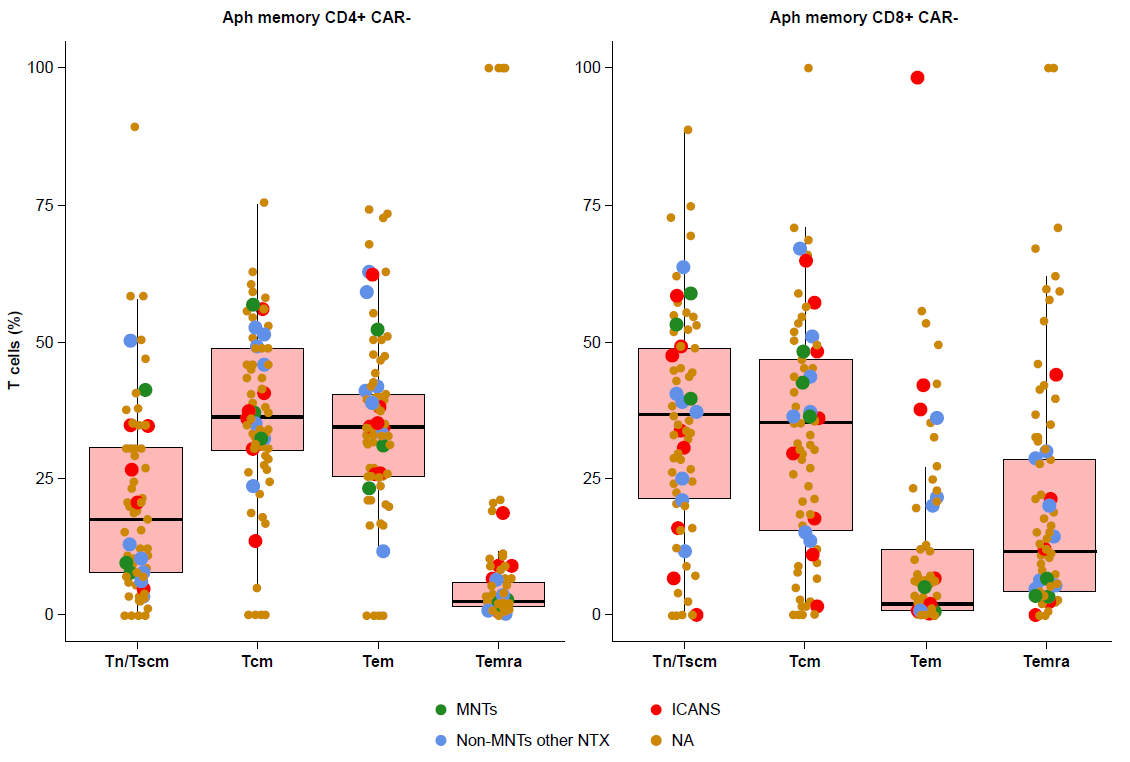


**Supplementary Fig. 4** Comparison of total number of CAR+ viable T cells administered with body weight normalization (**A**) and without body weight normalization (**B**) in patients with and without other CAR T-cell neurotoxicities in CARTITUDE-1. *CAR* chimeric antigen receptor.


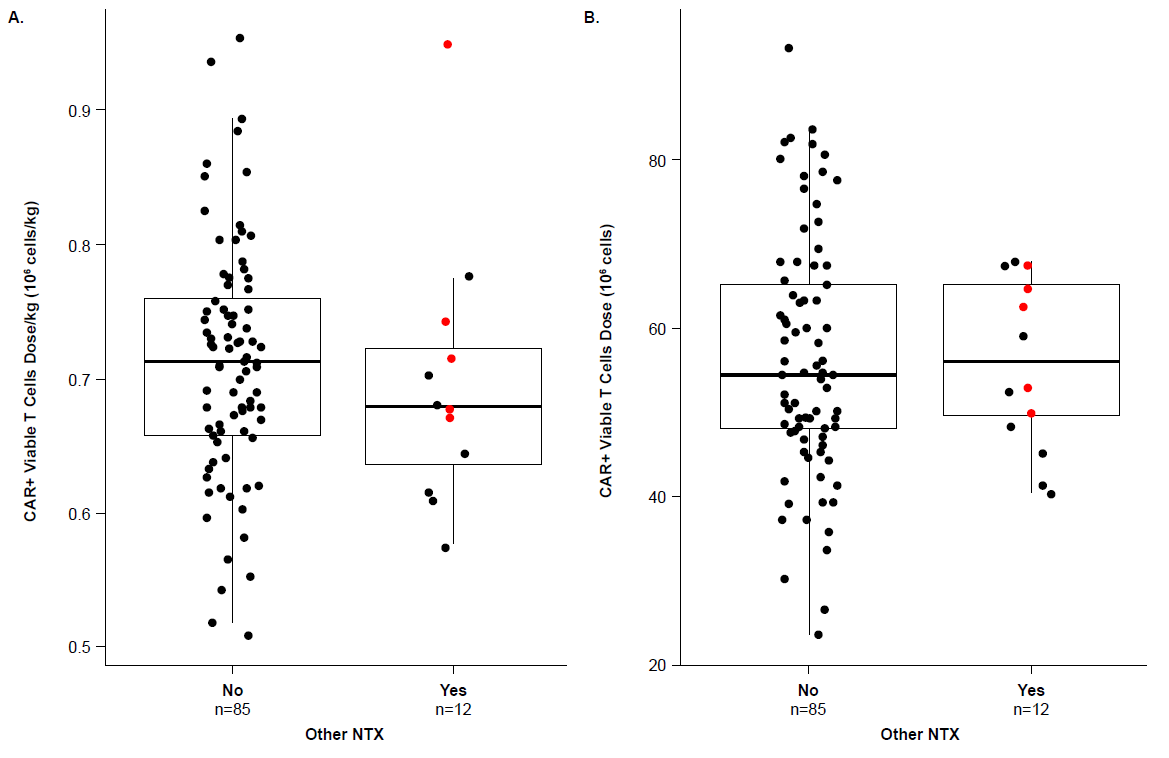

Supplement: Supplementary file 1 — Supplementary Appendix [file 41408_2022_629_MOESM1_ESM.docx]
